# Beta-Blocker Usage in Patients With Heart Failure With Reduced Ejection Fraction During Acute Decompensated Heart Failure Hospitalizations

**DOI:** 10.31486/toj.24.0011

**Published:** 2024

**Authors:** Matthew T. Brennan, Khaled M. Harmouch, Jawad Basit, M. Chadi Alraies

**Affiliations:** ^1^ Wayne State University School of Medicine, Detroit, MI; ^2^ Department of Internal Medicine, Wayne State University School of Medicine, Detroit Medical Center, Detroit, MI; ^3^ Department of Medicine, Rawalpindi Medical University, Rawalpindi, Punjab, Pakistan; ^4^ Cardiovascular Institute, Wayne State University School of Medicine, Detroit Medical Center, Detroit, MI

**Keywords:** *Adrenergic beta-antagonists*, *heart failure*, *heart failure–systolic*, *hospitalization*

## Abstract

**Background:** Acute decompensated heart failure accounts for more than 1 million hospitalizations in the United States every year. Beta-blockers are a first-line agent for patients experiencing heart failure with reduced ejection fraction, but beta-blocker use in patients hospitalized for acute decompensated heart failure remains low. We conducted an analysis of the existing evidence and guidelines to determine the conditions for prescribing beta-blockers to patients with acute decompensated heart failure.

**Methods:** We searched the PubMed database for studies from 2004 to 2024 that included the search terms “beta blockers” and “acute decompensated heart failure.” We included studies in which beta-blockers were used in patients with heart failure with reduced ejection fraction and excluded studies that did not study beta-blockers directly. We compiled recommendations from professional societies regarding beta-blocker usage—both for outpatients with heart failure with reduced ejection fraction and for patients hospitalized with acute decompensated heart failure.

**Results:** Studies consistently demonstrated lower rates of mortality and rehospitalization when beta-blocker therapy was maintained for patients with heart failure with reduced ejection fraction who were already on beta-blocker therapy. Conversely, withdrawal of beta-blocker therapy was associated with increased in-hospital and short-term mortality. We summarized our findings in a guideline-based flowchart to help physicians make informed decisions regarding beta-blocker therapy in patients with acute decompensated heart failure. Based on the evidence, beta-blockers should be initiated at a low dose in patients with heart failure with reduced ejection fraction who have never been on beta-blockers, provided the patient is hemodynamically stable.

**Conclusion:** Our research and our guideline-based flowchart promote guideline-directed use of beta-blockers to improve the outcomes of patients with heart failure with reduced ejection fraction.

## INTRODUCTION

As the population ages, heart failure is an increasing challenge in the United States. Hospitalizations for heart failure have been increasing since 2012.^1^ In 2017 alone, 924,000 Americans were hospitalized for heart failure, accounting for a total of 1.2 million hospitalizations.^1^

Heart failure is classified into 3 phenotypes based on left ventricular ejection fraction: heart failure with reduced left ventricular ejection fraction (≤40%); heart failure with mildly reduced left ventricular ejection fraction (41%-49%); and heart failure with preserved left ventricular ejection fraction (≥50%). In developed countries, patients with reduced ejection fraction account for up to 60% of patients with heart failure, while patients with mildly reduced or preserved ejection fraction make up the remaining 40%.^2,3^

Acute decompensated heart failure is generally defined as the gradual or sudden onset of new or worsening signs and symptoms of heart failure, leading to a hospitalization or emergency department visit.^4^ A 2005 report from Gheorghiade et al categorized patients by heart failure chronicity.^5^ Among the patients hospitalized for heart failure, approximately 70% represented worsening chronic heart failure, 25% represented new-onset heart failure, and 5% represented advanced heart failure.^5^

## METHODS

Because beta-blocker usage in patients hospitalized for acute decompensated heart failure is low, we conducted an analysis of the existing evidence and guidelines to determine the conditions for prescribing beta-blockers to patients with acute decompensated heart failure. We compiled recommendations from professional societies regarding beta-blocker usage both for outpatients with heart failure with reduced ejection fraction and for patients hospitalized with acute decompensated heart failure. We also searched the PubMed database for studies from 2004 to 2024 that included the search terms “beta blockers” and “acute decompensated heart failure” and included studies in which beta-blockers were used to treat patients with heart failure with reduced ejection fraction. We excluded studies that did not study beta-blockers directly.

## OUTPATIENT USE OF BETA-BLOCKERS FOR HEART FAILURE WITH REDUCED EJECTION FRACTION

Beta-blockers are a first-line treatment for heart failure with reduced ejection fraction. By antagonizing catecholamines at beta receptors, beta-blockers reduce the harmful effects of the chronic sympathetic activation seen in heart failure with reduced ejection fraction: increased heart rate, high myocardial oxygen demand, arrhythmogenicity, and cardiac remodeling. In large randomized controlled trials, 3 different beta-blockers—bisoprolol, carvedilol, and sustained-release metoprolol succinate—were shown to reduce all-cause mortality and hospitalization.^6-8^ Consequently, professional societies universally recommend beta-blockers as the initial treatment for patients with heart failure with reduced ejection fraction (Table 1).^4,9-11^

**Table 1.** Professional Society Recommendations on the Use of Beta-Blockers for Heart Failure With Reduced Ejection Fraction

Professional Society	Recommendation	Strength of Recommendation	Quality of Evidence
AHA/ACC/HFSA^9^	In patients with heart failure with reduced ejection fraction, with current or previous symptoms, the use of 1 of the 3 beta-blockers proven to reduce mortality (eg, bisoprolol, carvedilol, sustained-release metoprolol succinate) is recommended to reduce mortality and hospitalizations.	Class 1 (Strong)	Level A (multiple RCTs or meta-analyses)
ESC^4^	A beta-blocker is recommended for patients with stable heart failure with reduced ejection fraction to reduce the risk of heart failure hospitalization and death.	Class 1 (Strong)	Level A (multiple RCTs or meta-analyses)
CCS/CHFS^10^	We recommend that beta-blockers be initiated as soon as possible after the diagnosis of heart failure, including during the index hospitalization, provided that the patient is hemodynamically stable.	Strong	High
NICE^11^	First-line treatment: Offer an angiotensin-converting enzyme inhibitor and a beta-blocker licensed for heart failure to people who have heart failure with reduced ejection fraction. Use clinical judgment when deciding which drug to start first.	N/A	N/A

AHA/ACC/HFSA, American Heart Association/American College of Cardiology/Heart Failure Society of America; CCS/CHFS, Canadian Cardiovascular Society/Canadian Heart Failure Society; ESC, European Society of Cardiology; N/A, not available; NICE, National Institute for Health and Care Excellence; RCTs, randomized controlled trials.

## ACUTE DECOMPENSATED HEART FAILURE

### Presentation of Acute Decompensated Heart Failure

Patients presenting with acute decompensated heart failure require immediate medical evaluation and treatment. In-hospital mortality rates for acute decompensated heart failure hospitalizations range from 4.2% to 5.3%, increasing to 22% to 27% within 1 year of hospitalization.^12,13^ However, most presentations of decompensated heart failure are not acute. Instead, patients typically present to the hospital following a gradual increase in cardiac filling pressures coupled with a precipitating event.^9^ The most common precipitating factors in descending order are pneumonia or respiratory processes, arrhythmias, medication noncompliance, renal failure, and uncontrolled hypertension.^14^

To aid in the management of acute decompensated heart failure, patients are assigned to 1 of 4 categories depending on whether they show signs of congestion (ie, *wet* vs *dry*) or hypoperfusion (ie, *cold* vs *warm*).^15^ A study of 7,865 patients in the European Heart Failure Long-Term Registry found that patients most often fell into the warm-wet classification (70%), followed by cold-wet (20%), warm-dry (10%), and cold-dry (0.4%).^13^ Of note, hypotension (systolic blood pressure <90 mm Hg) is not necessary to diagnose hypoperfusion; only 5% to 8% of patients with acute decompensated heart failure present with hypotension.^15^ Instead, clinicians rely on clinical signs such as cold extremities, oliguria, or altered mental status.

### Management of Acute Decompensated Heart Failure

Management of patients with acute decompensated heart failure should focus on addressing reversible factors, optimizing volume status, and prescribing guideline-directed medical therapy.^9^ Optimization of volume status entails the use of loop diuretics to relieve congestion.^16^ In rare instances—such as a patient with low systolic blood pressure, hypoperfusion, and heart failure with reduced ejection fraction—inotropes may be required.^17^

As shown in Table 1, beta-blockers are a mainstay of guideline-directed medical therapy in patients with heart failure with reduced ejection fraction. Accordingly, professional societies recommend continuing or initiating beta-blockers during a hospitalization for acute decompensated heart failure in most situations (Table 2).^4,9-11^ We have created a guideline-based flowchart to help physicians determine when or whether to withdraw beta-blockers during a hospitalization for acute decompensated heart failure (Figure).

**Table 2.** Professional Society Recommendations on the Use of Beta-Blockers During a Hospitalization for Acute Decompensated Heart Failure

Professional Society	Recommendation	Strength of Recommendation	Quality of Evidence
AHA/ACC/HFSA^9^	In patients with heart failure with reduced ejection fraction requiring hospitalization, preexisting guideline-directed medical therapy should be continued and optimized to improve outcomes unless contraindicated.	Class 1 (Strong)	Level B-NR (nonrandomized trials)
ESC^4^	It is recommended that evidence-based oral medical treatment be administered before discharge.	Class 1 (Strong)	Level C
CCS/CHFS^10^	We recommend that beta-blockers be initiated as soon as possible after the diagnosis of heart failure, including during the index hospitalization, provided that the patient is hemodynamically stable. Clinicians should not wait until hospital discharge to start beta-blocker treatment in stabilized patients.	Strong	High
NICE^11^	In a person presenting with acute heart failure who is already taking beta-blockers, continue the beta-blocker treatment unless they have a heart rate less than 50 beats per minute, second or third-degree atrioventricular block, or shock.	N/A	N/A
	Start or restart beta-blocker treatment during hospital admission in people with acute heart failure due to left ventricular systolic dysfunction once their condition has been stabilized—for example, when intravenous diuretics are no longer needed.		

AHA/ACC/HFSA, American Heart Association/American College of Cardiology/Heart Failure Society of America; CCS/CHFS, Canadian Cardiovascular Society/Canadian Heart Failure Society; ESC, European Society of Cardiology; N/A, not available; NICE, National Institute for Health and Care Excellence.

**Figure.**
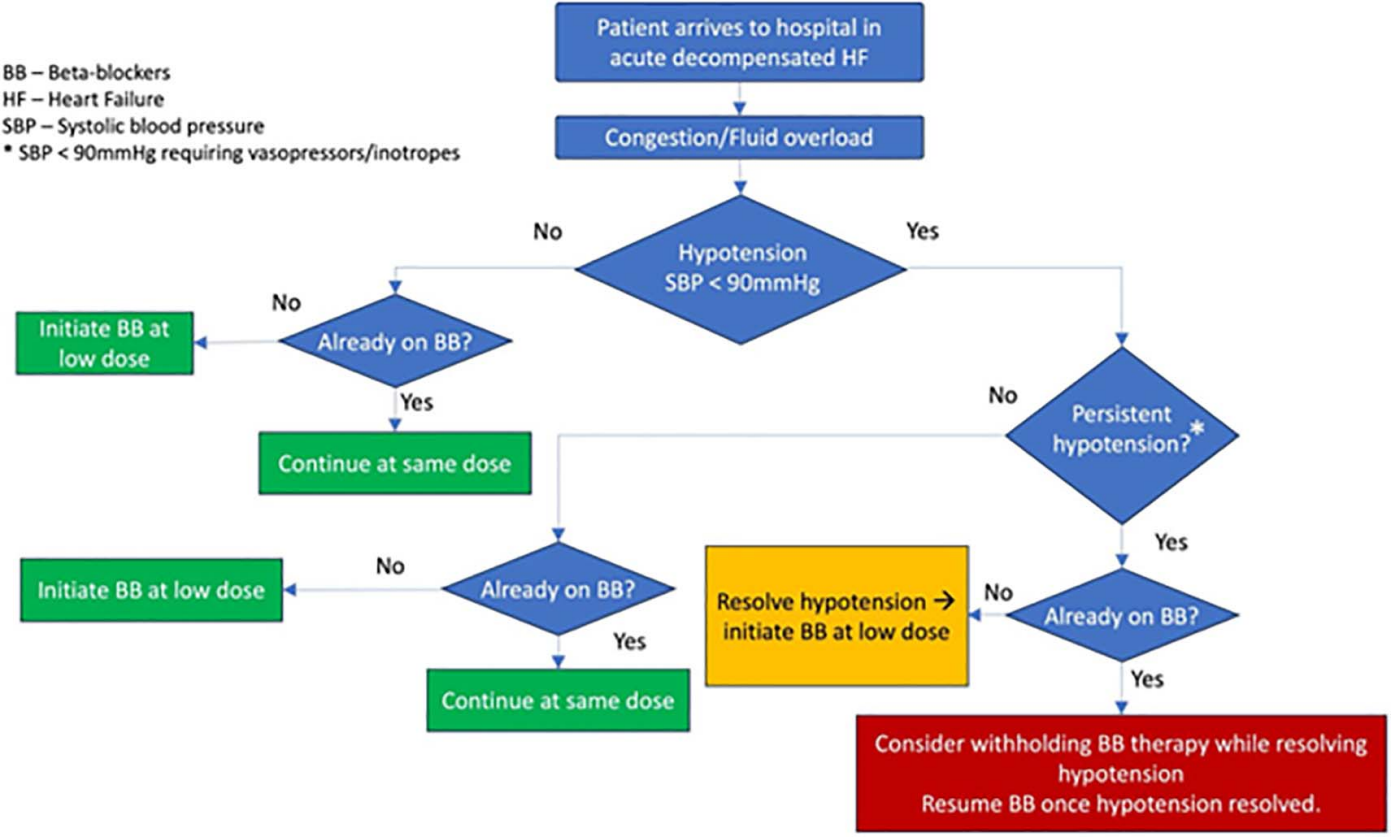
Guideline-based flowchart for using beta-blockers in patients with heart failure with reduced ejection fraction who present with acute decompensated heart failure.

The other components of guideline-directed medical therapy include renin-angiotensin system inhibitors, mineralocorticoid receptor antagonists, and sodium-glucose cotransporter 2 inhibitors.^9^ Greene et al analyzed medications taken by patients with heart failure with reduced ejection fraction in the Change the Management of Patients with Heart Failure (CHAMP-HF) registry and found that the guideline-directed medical therapy medications most often reduced in dosage were angiotensin-converting enzyme inhibitors/angiotensin II receptor blockers and beta-blockers.^18^

Physicians may have concerns about continuing a medication with negative inotropic effects at a time of worsening hemodynamics.^19^ This instinct may make sense in theory but does not hold in practice for several reasons. First, the initial goal in the management of acute decompensated heart failure is to address reversible factors. Beta-blocker medications would be a suspected culprit of decompensation only if they were initiated or increased shortly before the hospitalization. If not, physicians should look elsewhere for reversible factors precipitating an acute decompensated heart failure hospitalization. Second, sudden cessation of beta-blocker treatment may cause further adverse outcomes by causing a rebound of the sympathetic nervous system.^20^ Beta-blocker withdrawal has been shown to increase angina and the risk of coronary events.^21^

Despite the reasoning for the continuation of beta-blocker therapy during hospitalization for acute decompensated heart failure, many providers fail to administer them. In a 2018 analysis of the CHAMP-HF registry, just 67% of patients with heart failure with reduced ejection fraction were prescribed beta-blockers.^22^ An analysis of claims data from 2009 to 2012 found that 42% of patients with heart failure with reduced ejection fraction were not prescribed any heart failure–specific medications as of 30 days following hospitalization.^23^ Although hypoperfusion is observed in only about 1 in 5 patients with acute decompensated heart failure,^13^ physicians often cited “medical reasons” as the impetus for reducing dosages of beta-blocker therapy.^18^

## IN-HOSPITAL MANAGEMENT GUIDELINES

### Withdrawal of Beta-Blocker Therapy During Hospitalization Is Harmful

The withdrawal of beta-blockers in patients with heart failure with reduced ejection fraction experiencing acute decompensated heart failure is harmful in the short and long term. In the Organized Program to Initiate Lifesaving Treatment in Hospitalized Patients With Heart Failure (OPTIMIZE-HF), patients whose beta-blocker treatment was withdrawn had higher rates of mortality compared to patients who continued beta-blocker treatment.^24^ A 2015 meta-analysis found beta-blocker discontinuation to be associated with an increased risk of both in-hospital mortality and short-term mortality (≤6 months of first hospitalization).^19^ Beta-blocker discontinuation also led to a higher risk of the combined outcome of rehospitalization or mortality.^19^

### Maintenance of Beta-Blocker Therapy Is Beneficial

Studies support the claim that continuation of beta-blocker therapy throughout hospitalization leads to significant benefits. The OPTIMIZE-HF trial found reductions in 60-day mortality, 90-day mortality, and the combined outcome of rehospitalization or mortality in patients discharged on carvedilol compared to patients not on carvedilol.^25^

One benefit of continuing beta-blocker therapy throughout hospitalization may be the higher rate of adherence upon discharge, given the difficulty of resuming beta-blockers once they are discontinued. Higher usage of beta-blockers has been observed in patients whose medication was not discontinued during an acute decompensated heart failure hospitalization.^26^ The benefit of maintaining beta-blocker therapy during hospitalization may be associated with the difficulty patients face in reaching target dosages. In the CHAMP-HF registry, just 20.3% of patients with heart failure with reduced ejection fraction were prescribed target doses of beta-blockers at baseline, improving to only 26.4% after 12 months.^18^ The suboptimal beta-blocker dosage prescriptions may be due to the “start low, go slow” approach recommended when prescribing beta-blockers.^27^ Given the time needed to achieve optimal dosages, the choice to withdraw or decrease beta-blocker therapy during hospitalization takes on greater importance. A single hospitalization has the potential to undo months or years of careful titration.

### Initiation of Beta-Blocker Therapy Is Recommended in Beta-Blocker–Naïve Patients

Beta-blocker–naïve patients should be started on beta-blocker therapy during an acute decompensated heart failure hospitalization because initiation during hospitalization consistently benefits patients. A 2018 analysis of 672 beta-blocker–naïve patients who were hospitalized for acute decompensated heart failure and received inotropes showed that the predischarge initiation of beta-blockers resulted in lower 2-year mortality (21.3% vs 39.3%).^28^ The authors also found that predischarge prescriptions of beta-blockers in beta-blocker–naïve patients resulted in significantly higher prescription rates after 12 months (89% vs 25%).^28^ In an analysis of OPTIMIZE-HF, Fonarow et al found that initiation of beta-blockers during a heart failure hospitalization was well tolerated, as evidenced by 91.9% of patients maintaining therapy at 60 to 90 days postdischarge follow-up.^24^

Professional societies also recommend initiating beta-blocker therapy during acute decompensated heart failure hospitalizations in beta-blocker–naïve patients. The 2021 European Society of Cardiology (ESC) guidelines state, “In patients admitted with AHF [acute heart failure], beta-blockers should be cautiously initiated in hospital, once the patient is haemodynamically stabilized.”^4^ The 2022 American Heart Association/American College of Cardiology/Heart Failure Society of America (AHA/ACC/HFSA) guidelines concur: “Oral GDMT [guideline-directed medical therapy] should not be withheld for mild or transient reductions in blood pressure.” The AHA/ACC/HFSA guidelines continue, “True contraindications are rare, such as advanced degree atrioventricular block….”^9^

Despite these encouraging findings, physicians should carefully monitor symptoms when initiating beta-blocker therapy. The Metoprolol CR/XL Randomised Intervention Trial in Congestive Heart Failure (MERIT-HF) found that the most common limiting factor for reaching target dosages of metoprolol CR/XL in the outpatient setting was low heart rate, seen in 11.3% of patients.^29^ MERIT-HF also found that the greatest risk for mortality or hospitalization occurred 4 to 8 weeks following initiation of beta-blockers. After 8 weeks, rates of deterioration shifted to favor beta blockade in all patients taking beta-blockers. Patients’ subjective well-being could even predict future events. Lainscak et al found that self-reported health was an independent predictor of adverse events in patients with chronic heart failure during the period of initiating or increasing beta-blocker dosages.^30^

### Consider Withholding Beta-Blocker Therapy in Patients With Persistent Hypotension

In patients with persistent hypotension, physicians should consider withholding or reducing the dosage of beta-blocker therapy. To our knowledge, no studies have compared continuation vs withdrawal of beta-blocker therapy in acute decompensated heart failure patients with unresolved hypotension. However, professional guidelines recommend that physicians consider withholding beta-blockers or reducing the dosage while resolving persistent hypotension. The 2021 ESC guidelines state, “In those admitted with ADHF [acute decompensated heart failure], oral OMT [optimal medical therapy] should be continued, except for possible dose reduction or withdrawal if there is haemodynamic instability (symptomatic hypotension), severely impaired renal function or hyperkalaemia. Once haemodynamic stabilization is achieved with i.v. therapy, treatment should be optimized before discharge.”^4^ The 2022 AHA/ACC/HFSA guidelines state, “Withholding or reducing beta-blocker therapy should be considered in patients with marked volume overload or marginal low cardiac output.”^9^

## CONCLUSION

Our analysis underscores the critical role of beta-blocker therapy in the treatment of patients with heart failure with reduced ejection fraction who present with acute decompensated heart failure. The difficulty in attaining optimal beta-blocker dosages in the outpatient setting amplifies the importance of maintenance therapy during hospitalization. Discontinuing beta-blocker therapy during episodes of acute decompensated heart failure is associated with adverse outcomes in both the short and long term. Beta-blockers should be continued in patients already on stable regimens, except in rare situations. Additionally, for patients not previously on beta-blocker therapy, initiation should occur as soon as the patient is hemodynamically stable. The guideline-based flowchart we provide serves as a guide for physicians in making informed decisions regarding beta-blocker therapy for patients with heart failure with reduced ejection fraction who are admitted to the hospital with acute decompensated heart failure.

## Notes

*This article meets the Accreditation Council for Graduate Medical Education and the American Board of Medical Specialties Maintenance of Certification competencies for Patient Care, Medical Knowledge, and Practice-Based Learning and Improvement.*
